# Supplementary material for: Decoding emergent properties of microbial community functions through subcommunity observations and interpretable machine learning
Source: ISME J. 2025 Oct 23;19(1):wraf236. doi: 10.1093/ismejo/wraf236 (PMC12599306; doi:10.1093/ismejo/wraf236)
Supplement: Supplementary_Materials_wraf236 [file supplementary_materials_wraf236.pdf]

Supplementary Materials

Decoding emergent properties of microbial community functions through sub-community observations and interpretable machine learning

Hidehiro Ishizawa[1*], Sunao Noguchi[1], Miku Kito[1], Yui Nomura[2], Kodai Kimura[2], Masahiro Takeo[1]

[1]Department of Applied Chemistry, Graduate School of Engineering, University of Hyogo, Himeji, Hyogo, 671-2280, Japan

[2]Department of Applied Chemistry and Chemical Engineering, School of Engineering, University of Hyogo, Himeji, Hyogo, 671-2280, Japan

*Corresponding author

Hidehiro Ishizawa

Address: Shosha 2167, Himeji, Hyogo, 671-2280 Japan

Phone: (+81)79-267-4969

Mail to: ishizawahidehiro@gmail.com

Contents

- **Supplementary Note 1**
  Methods for 16S rDNA amplicon sequencing

- **Supplementary Note 2**
  Methods for calculation of area under the curve (AUC)

- **Supplementary Note 3**
  Methods for validation of fluorescence-based measurements

- **Supplementary Figure S1**
  Community composition of restored model aniline-degrading community

- **Supplementary Figure S2**
  Aniline-degrading ability of the nine synthetic community members

- **Supplementary Figure S3**
  Validation of fluorescence-based measurement of aniline-utilization activity and Cab074F growth.

- **Supplementary Figure S4**
  Composition–abundance landscape of strain Cab074F

- **Supplementary Figure S5**
  Correlation between functional effect of each non-degrader strain and functional level of the background community

- **Supplementary Table S1**
  Analytical conditions for HPLC

- **Supplementary Table S1**
  Identification results of synthetic community members

- **Supplementary Table S1**
  Quality, abundance, and taxonomic position of metagenome-assembled genomes

Supplementary Note 1

For amplicon sequencing, the 16S rRNA gene library (515F–806R) was prepared using a two-step PCR procedure and sequenced on the Illumina Miseq (2×300 bp) at Bioengineering Lab. Co. Ltd. (Sagamihara, Japan). TaKaRa Ex Premier DNA Polymerase (Takara Bio, Shiga, Japan) was used for PCR according to the manufacture's protocol. Sequencing reads were processed using DADA2 [1] to generate an ASV table, and taxonomy was assigned using the SILVA v138 database [2].

Supplementary Note 2

AUC was calculated using the R package "gcplyr" [3] as follows: (1) baseline correction by subtracting the mean relative fluorescence units (RFU) values of blank wells on the same plate; (2) alignment of time windows across plates by shifting to match the lag phase of the Cab074F-only wells, as estimated using gcplyr; (3) normalization of the RFU values by setting adjusted time 0 to 1 for each well; and (4) normalization of the AUC values by dividing the mean AUC of the Cab074F-only wells.

Supplementary Note 3

To validate that resorufin fluorescence reflected aniline-utilization capacity, cultivation assays for 18 randomly selected combinations were performed using the same procedure to the 384-well plate-based assay but scaled up to 300 µL in a 96-well plate to obtain sufficient samples for HPLC. Cultures were prepared in duplicate wells, and samples were collected after incubating 48 and 72 h to evaluate aniline degradation via HPLC. These results were compared with the RFU values of resorufin measured at the corresponding time points. Similarly, the correlation between GFP fluorescence and Cab074F abundance was validated by plating 13 Cab074F cultures in a 96-well plate after 24, 48, and 72 h on MSM aniline medium solidified with agarose and comparing Cab074F abundance in CFU/mL with the corresponding GFP RFU values.

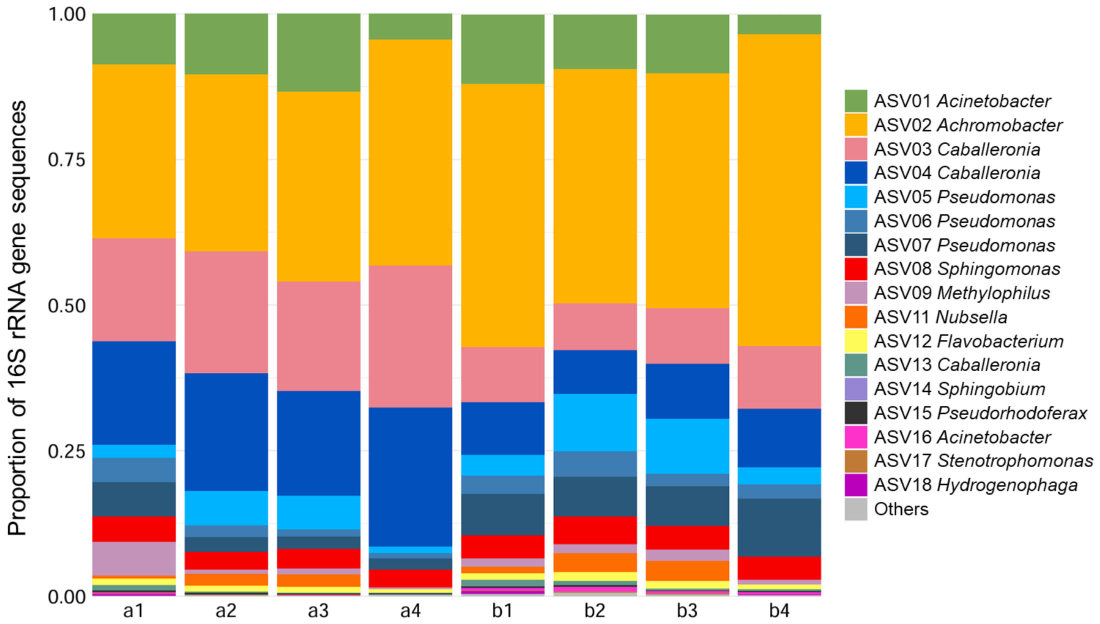

Fig. S1 Reproducibility of community composition in the restored model aniline-degrading community. The community was restored twice from glycerol stocks on different dates (labels "a" and "b" denote the two restoration dates), each in four replicates (see Materials and Methods). Community composition at 48 h was assessed by amplicon sequencing.

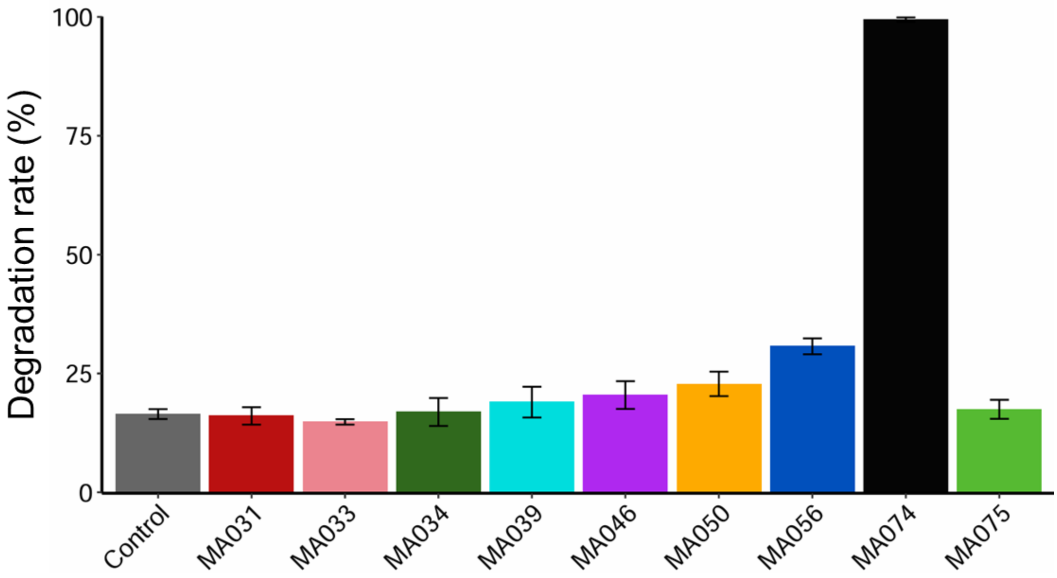

Fig. S2 Aniline-degrading ability of the nine synthetic community members. Degradation rates for 2 mM aniline over 48 h of cultivation are shown. The observed decrease in the uninoculated control reflects evaporation during cultivation. Error bars represent the standard deviation (n = 3).

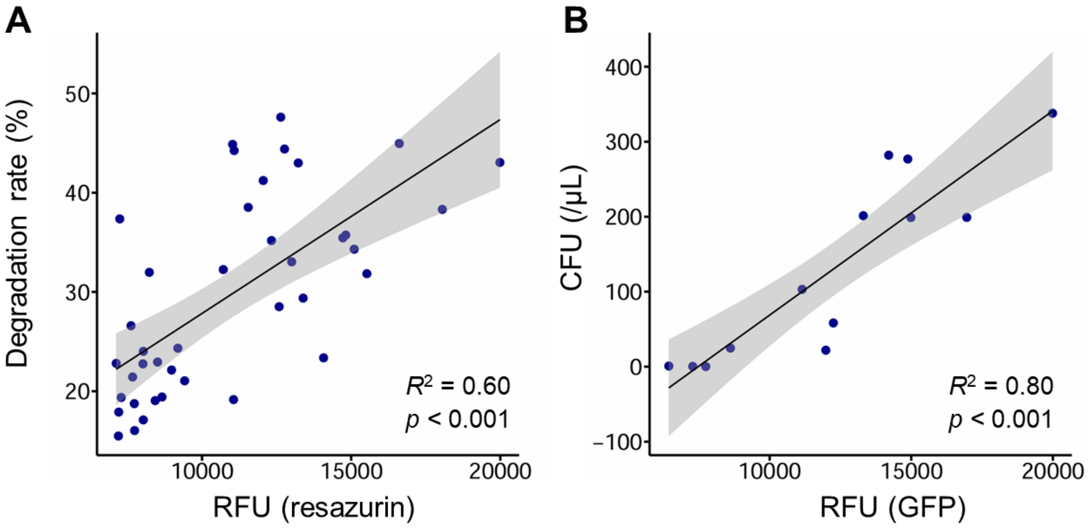

Fig. S3 Validation of fluorescence-based measurements of aniline-utilization activity and Cab074F growth. Selected microplate cultures were evaluated for resazurin RFU and aniline degradation rate, or GFP fluorescence and colony forming units of Cab074F; paired values were compared (see Materials and Methods). A) Correlation between resazurin fluorescence and aniline degradation rate. B) Correlation between GFP fluorescence and CFU of strain Cab074F.

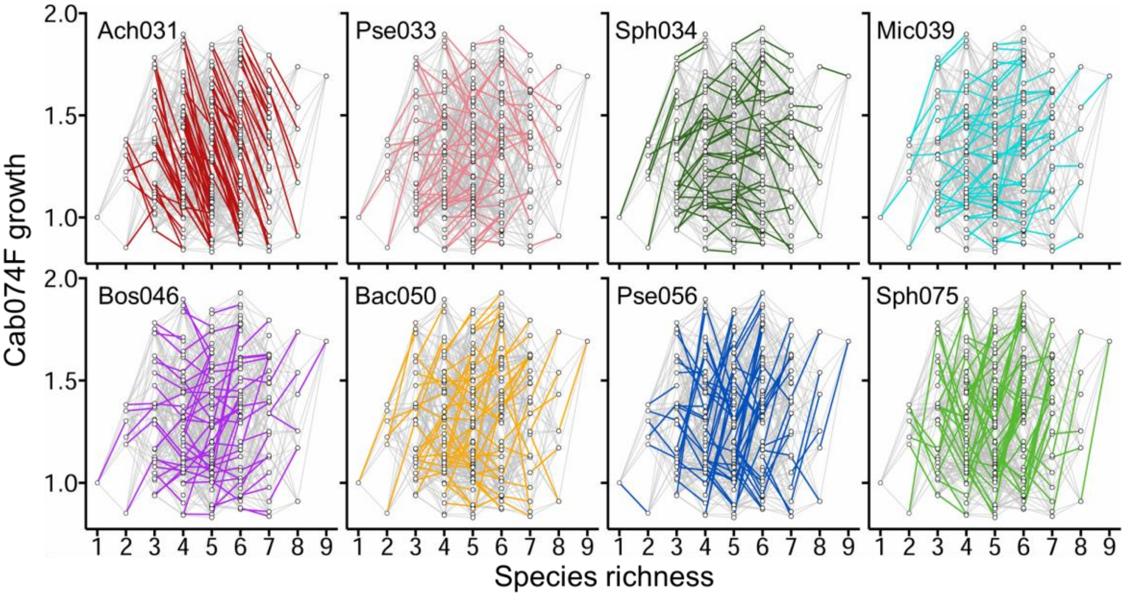

Fig. S4 Composition–abundance landscape constructed from the dataset of strain Cab074F's growth in the 256 sub-community combinations. Each data point represents a sub-community combination with a given species richness. The angles of the edges indicate the effect of each non-degrader strain on Cab074F growth in different community contexts.

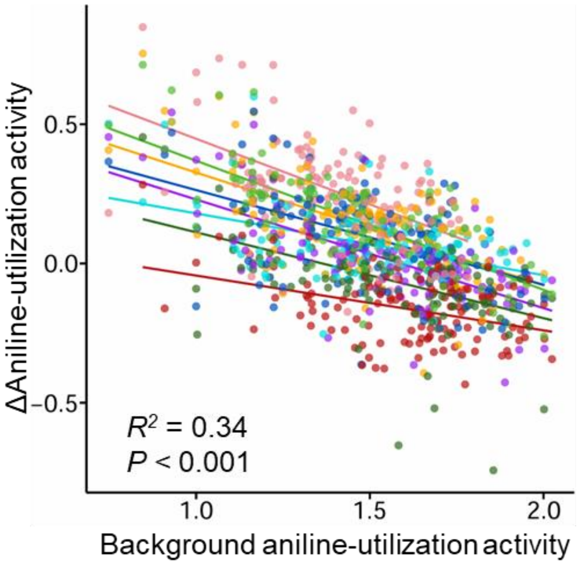

Fig. S5 Correlation between the functional effect of each non-degrader strain and the functional level of the background community. The change in aniline-utilization activity upon adding a non-degrader strain (Δaniline-utilization activity) was plotted against the aniline-utilization activity of the corresponding background subcommunity combinations. Colors as in Fig. S4.

Table S1 Analytical conditions for HPLC

Parameter	Operation conditions
Column	ODS-80Ts (Tosoh, Tokyo, Japan)
Mobile phase	CH_3CN:H_2O:CH_3COOH = 400:600:1
Detection wavelength	240 nm and 260 nm
Flow rate	0.5 mL/min
Injection volume	20 μL
Column temperature	40℃

Table S2 Identification results of synthetic community members based on 16S rDNA sequences. The closest MAG–isolate correspondence was predicted based on species-level identification results.

Strain	Most similar species	Top-hit strain	Similarity	Closest MAG	Accession
Ach031	*Achromobacter veterisilvae*	LMG30378	99.5%	MAG02	LC878797
Pse033	*Pseudomonas monteilii*	NBRC 103158	99.9%	MAG03	LC878798
Sph034	*Sphingomonas pseudosanguinis*	G1-2	99.4%	-	LC878799
Mic039	*Microbacterium laevaniformans*	DSM 20140	99.7%	MAG06	LC878800
Bos046	*Bosea spartocytisi*	SSUT16	99.9%	-	LC878801
Bac050	*Bacillus mexicanus*	FSQ1	99.8%	-	LC878802
Pse056	*Pseudomonas guguanensis*	JCM 18416	97.8%	MAG05	LC878803
Cab074	*Caballeronia cordobensis*	LMG 27620	99.7%	MAG01	LC878804
Sph075	*Sphingomonas cannabina*	DM2-R-LB4	98.3%	MAG04	LC878805

Table S3 Quality, abundance, and taxonomic placement of metagenome-assembled genomes (MAGs). Completeness and contamination were assessed with CheckM. Abundance refers to the proportion of metagenomic reads mapping to each MAG. The closest species and average nucleotide identity (ANI), computed with DFAST, are also shown.

ID	Completeness (%)	Contamination (%)	Abundance (%)	Closest species	ANI (%)
MAG01	94.8	0.4	37.8	*Caballeronia cordobensis*	97.7
MAG02	98.1	0.4	27.9	*Achromobacter denitrificans*	99.2
MAG03	80.7	0.0	9.9	*Pseudomonas monteilii*	96.6
MAG04	99.7	0.9	1.2	*Sphingomonas kyeonggiensis*	89.3
MAG05	97.0	0.6	0.7	*Pseudomonas insulae*	86.9
MAG06	99.5	1.0	0.5	*Microbacterium laevaniformans*	94.0
MAG07	94.4	0.7	0.5	*Pseudorhodoferax aquiterrae*	88.8
MAG08	91.8	0.6	0.4	*Stenotrophomonas nitritireducens*	92.3
MAG09	98.3	1.9	0.3	*Sphingobium fuliginis*	89.4
MAG10	89.0	0.0	0.2	*Methylophilus methylotrophus*	83.3
MAG11	87.3	0.0	0.2	*Methylophilus rhizosphaerae*	80.4
MAG12	51.7	0.0	0.1	*Acinetobacter pittii*	97.1
MAG13	84.0	0.7	0.1	*Microbacterium hydrocarbonoxydans*	82.9

References

1. Callahan BJ, McMurdie PJ, Rosen MJ et al. DADA2: High-resolution sample inference from Illumina amplicon data. *Nat Methods* 2016;**13**:581–3.
2. Quast C, Pruesse E, Yilmaz P et al.The SILVA ribosomal RNA gene database project: improved data processing and web-based tools. *Nucleic Acids Res* 2013;**41**:D590–6.
3. Blazanin M. gcplyr: an R package for microbial growth curve data analysis. *BMC Bioinform* 2024;**25**:232.
